# Healing Function for Abraded Fingerprint Ridges in Tactile Texture Sensors

**DOI:** 10.3390/s24134078

**Published:** 2024-06-23

**Authors:** Muhammad Irwan Yanwari, Shogo Okamoto

**Affiliations:** 1 Department of Computer Science, Tokyo Metropolitan University, Tokyo 191-0065, Japan; irwan.yanwari@polines.ac.id; 2 Department of Electrical Engineering, Politeknik Negeri Semarang, Kota Semarang 50275, Indonesia

**Keywords:** tactile sensor, self-healing material, abrasion damage

## Abstract

Tactile texture sensors are designed to evaluate the sensations felt when a human touches an object. Prior studies have demonstrated the necessity for these sensors to have compliant ridges on their surfaces that mimic human fingerprints. These features enable the simulation of contact phenomena, especially friction and vibration, between human fingertips and objects, enhancing the tactile sensation evaluation. However, the ridges on tactile sensors are susceptible to abrasion damage from repeated use. To date, the healing function of abraded ridges has not been proposed, and its effectiveness needs to be demonstrated. In this study, we investigated whether the signal detection capabilities of a sensor with abraded epidermal ridges could be restored by healing the ridges using polyvinyl chloride plastisol as the sensor material. We developed a prototype tactile sensor with an embedded strain gauge, which was used to repeatedly scan roughness specimens. After more than 1000 measurements, we observed significant deterioration in the sensor’s output signal level. The ridges were then reshaped using a mold with a heating function, allowing the sensor to partially regain its original signal levels. This method shows potential for extending the operational lifespan of tactile texture sensors with compliant ridges.

## 1. Introduction

Epidermal ridges, also known as dermatoglyphs, are raised, corrugated patterns on the skin’s surface, notably on the palms of the hands. These structures play a crucial role in human tactile sensing capabilities [1,2,3,4,5,6]. In surface textural sensing, the ridges generate vibrations when sliding over roughened surfaces, and the vibrations are transferred to mechanoreceptors inside the skin [4,5]. It has been empirically demonstrated that epidermal ridges can enhance tactile signals by a factor of 100 in response to vibrations induced by textures of specific wavelengths [6]. Many studies in tactile sensors have attempted to replicate the human finger structure to emulate the sensing function of the human finger pad [7,8,9,10,11,12,13,14]. For example, Lu et al. [9] replicated a sensor with a multi-layered structure that comprises upper layers characterized by high sensitivity to emulate tactile functionality alongside lower layers featuring an extensive measurement range to facilitate pressure sensing. Kim et al. [10] designed a sensor with a ridged structure found in different parts of a human fingerprint.

The absence of epidermal ridges significantly affects the signal from texture sensors. Human epidermal ridges possess a healing function that helps to maintain their shape after minor injuries [15,16]. Unlike human sensors, synthetic tactile sensors do not inherently possess mechanisms to maintain their structural integrity. To address the damage of sensor or robotic skin, researchers have explored strategies to emulate the regenerative capacities of human skin, including the healing from cuts using healable materials [17,18,19,20,21,22,23,24,25,26]. For example, Roels et al. [19,20] employed thermo-reversible Diels–Alder bonds to manufacture a soft finger that can heal from cuts through heating. The finger was equipped with a bending sensor circuit made from the healable material. Tee et al. [17] demonstrated a synthetic skin with a self-healing function for cuts. This skin material was conductive and exhibited pressure-sensitive performance. Khatib et al. [26] designed sensor circuits for measuring the pressure, pH, and temperature on a self-healing material. The material’s healing function was effective at room temperature and was further enhanced by heating. Zhou et al. [27] developed a healable fiber with piezoresistive properties that can be used for knitting cloth with strain-sensing capability. However, to date, healing materials have not been used for tactile texture sensors with epidermal ridges to enhance the measurement of surface asperity.

This study focuses on restoring tactile texture sensors affected by abrasion damage, specifically targeting the epidermal ridges segment of the sensor. To date, no studies have validated healing functions for the surface ridges of tactile texture sensors. Further, thus far, no such healing method has been implemented. Abrasion damage, which involves the removal of material from the sensor surface, displays distinct characteristics compared to cut damage. The missing portions are often very small, making reattachment, such as in the method described by Wang et al. [28], impractical. To address this challenge, our approach involves using a reshapeable material as the primary component of the tactile sensor. This material is complemented by a heating mold that facilitates the reshaping of the damaged epidermal ridges. We prototyped a tactile texture sensor using this material and tested its performance by sliding it over roughness specimens with designed surface spatial wavelengths. The objective of this study is to demonstrate the effectiveness of the sensor’s healing function by recording the changes in the transducer’s outputs before and after abrasion and healing, as well as to describe the method used to heal the abraded sensor’s ridges.

## 2. Sensor Design and Manufacturing

### 2.1. Structure and Dimensions

**Figure 1:**
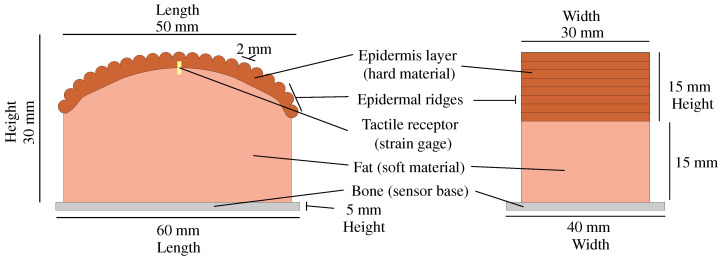
Sensor design. (**Left**): front view. (**Right**): side view.

We devised a texture sensor, as illustrated in Figure 1, with a structure similar to those manufactured in previous studies [29,30,31]. The sensor was constructed with two layers mirroring the composition of human skin’s epidermal and fat layers. The inner soft layer exhibited a hardness of 0.17 MPa, while the outer hard layer had a hardness of 0.82 MPa. These values are close to those determined by an anatomical study [32].

The designed sensor had dimensions of 50 mm in length, 30 mm in width, and 30 mm in height. The sensor’s plastic base had dimensions of 60 mm in length, 40 mm in width, and 5 mm in height. The top section of the sensor was circular, with a diameter of 65 mm, imitating the shape of a human finger pad. During preliminary experiments, an alternative sensor with a flat top surface was also employed. However, the edge of this sensor made intensive contact with target specimens, posing challenges in maintaining secure contact during sliding. The sensor weighed 70 g, including the plastic base, strain gauge, and its cable.

Atop the epidermal layer, a raised pattern was used to emulate the functionality of epidermal ridges. This raised pattern was semi-circular with a diameter of 2 mm and had a thickness of 2 mm beneath the ridge within the epidermal layer. The diameter of the ridge is four to five times larger than that of an adult finger [33], necessitating downsizing in future iterations. The patterned outer layer covered the top half of the sensor.

The most prominent natural frequency of the sensor was 415 Hz, as determined by the peak frequency of the free vibration measured by the embedded transducer described in Section 2.3.

### 2.2. Material

As the material with healing function, we employed polyvinyl chloride plastisol (Plastic Worm, Two-L Co., Ltd., Shiogama, Japan), of which hardness can be modulated by adjusting the mixing ratio of softener and hardener solutions. As mentioned in Section 2.4, the material becomes loosened above 70 °C and completely liquefied at approximately 180 °C.

In order to determine hardness of each layer, we employed a formula from Gent [34,35] regarding the relation between indentation hardness or Shore hardness and Young’s modulus. We used a durometer (GS-721N, Teclock Co., Ltd., Okaya, Japan) for measuring Shore hardness of the materials.

### 2.3. Transducer

The tactile sensor incorporated a strain gauge (KFGS-1-120-C1-11 L3M2R, Kyowa Electronic Instruments Co., Ltd., Chofu, Japan) as a transducer to emulate the sensory functionality of human tactile receptors. The strain gauge was 1 mm in length, with a gauge factor of 2.10 and a resistance of 119.6 Ω. The strain gauge was positioned in the middle between the inner and outer layers beneath the central ridge, as shown in Figure 1. The signals from the strain gauge were conditioned by a dynamic strain amplifier (DPM-913B, Kyowa Electronic Instruments Co., Ltd., Chofu, Japan). We set the strain gauge configuration to 1500 με/V. Data collection involved a data acquisition device (USB-6002, National Instruments Co., Austin, TX, USA) controlled by the *Data Acquisition Toolbox* in MATLAB (2023b, MathWorks Co., Natick, MA, USA).

### 2.4. Sensor Manufacturing

**Figure 2:**
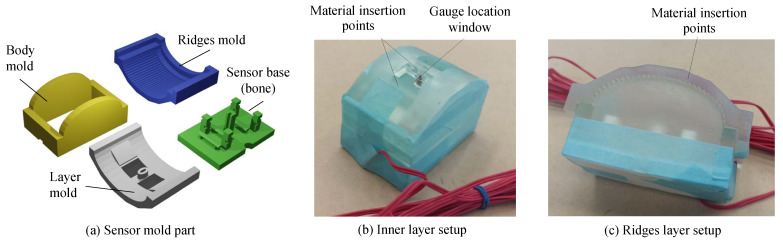
Sensor molds. (**a**) Four types of molds. (**b**) Photo of assembled molds for inner layer. (**c**) Photo of assembled molds for outer layer.

To manufacture the sensor, plastic molds created using the stereolithography technique (Form 3+, Formlabs, Somerville, MA, USA) were employed. These molds consisted of four primary components, as shown in Figure 2a. The sensor base, a part of the sensor, features a central pillar designed to secure the cable of the strain gauge and prevent its displacement during the manufacturing process. Additionally, four peripheral pillars on the sensor base functioned as adhesive anchors for the soft material, preventing separation between the plastisol and the sensor base during use. The body mold contained the liquid plastisol during the manufacturing process of the inner layer. Tape was used to seal the gap between the molds, as illustrated in Figure 2b,c. The layer mold secured the position of the strain gauge between the inner and outer layers while maintaining the semi-circular shape of the inner layer. During the formation of the inner layer, liquid plastisol was poured through two holes located at the top of the layer mold. After completing the inner layer, the manufacturing process was paused for two hours to allow for the solidification of the inner layer before proceeding to the epidermal layer. Following this interim period, the layer mold was removed from the assembly and replaced with the ridge mold. The filling process for the ridge mold was executed from the side of the sensor. Subsequently, the sensor underwent a two-hour cooling period before being extracted from the entire mold assembly.

### 2.5. Healing Processes

**Figure 3:**
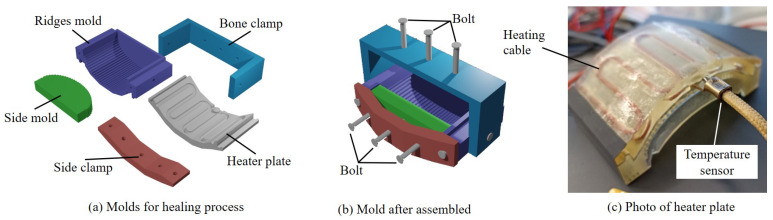
Molds for the healing process. (**a**) Five types of molds used for the healing process. (**b**) Assembly of the molds. (**c**) Photo of the heater plate, cable, and temperature sensor.

The sensor utilized a non-autonomous healing mechanism, specifically heating the material for reshaping. The surface of the abraded tactile sensor was first cleaned with an industrial paper towel. The sensor was then placed within a set of molds consisting of five parts, as shown in Figure 3a. These molds were fabricated using a heat-resistant resin (High Temp Resin, Formlabs, Somerville, MA, USA).

The heater plate, connected to a temperature controller (TXN-200AL, As One Co., Ltd., Osaka, Japan), was affixed to the ridge mold. The heating cable and temperature sensor were positioned between the heater plate and the ridge mold (Figure 3c). Heating the plate initiated the gelling of polyvinyl chloride plastisol. The ridge mold replicated the exact surface ridge patterns used in the manufacturing process, while the side mold prevented the leakage of liquefied material. Side and bone clamps applied pressure to the sensor from two separate directions.

During the healing process, the ridge mold and heater plate were heated to 70 °C for five minutes. Although the material is known to completely liquefy at 180 °C, a lower temperature is sufficient to turn the material into a gel. This gel fits into the female ridge pattern of the mold under applied pressure. Additionally, heating at a temperature below 100 °C prevents the formation of bubbles or vaporization of water content within the material. Furthermore, this low temperature helps to retain the position of the strain gauge within the tactile sensor. The strain gauge and plastisol are not bonded; thus, if the plastisol is overly heated, the gauge’s position may shift in the liquefied material (also see the fifth paragraph in Section 5).

After heating, the sensor was retained within the healing mold until the mold temperature had sufficiently decreased before extraction. The sensor could be easily removed from the molds because the surface of the polyvinyl chloride plastisol was oily immediately after reshaping. Thus, no lubricant oils were used on the molds.

## 3. Experiment

### 3.1. Textural Specimens

**Figure 4:**
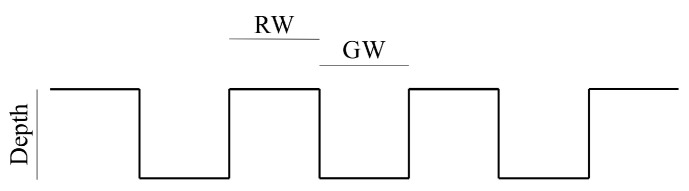
Specimen configuration. Ridge width (RW) and groove width (GW) were equal and they were 0.5 for λ = 1 mm or λ = 2 mm.

**Figure 5:**
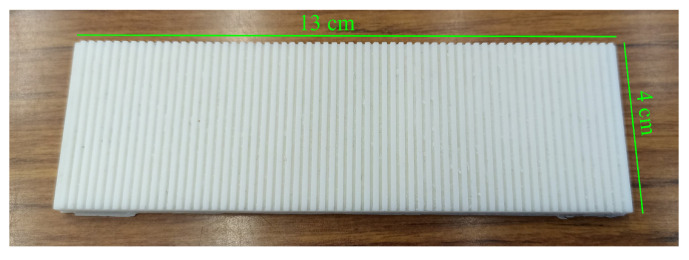
Photo of the grating scale with λ = 2 mm (RW and GW = 1 mm). The depth of the groove was 1 mm. The length and width were 13 cm and 4 cm, respectively.

The specimens scanned by the tactile sensor were grating scales featuring alternating grooves and ridges, as shown in Figure 4. The ratio of ridge width (RW) to groove width (GW) was 1:1, and the surface wavelength λ was defined as RW + GW. The specimens were composed of plastic (White Resin, Formlabs, Somerville, MA, USA) and had a total length of 13 cm, as depicted in Figure 5. We used two types of grating scales: one with RW and GW both at 1 mm, and another with RW and GW both at 0.5 mm. The depth of the grooves was equal to the corresponding RW and GW values.

### 3.2. Scanning Condition

**Figure 6:**
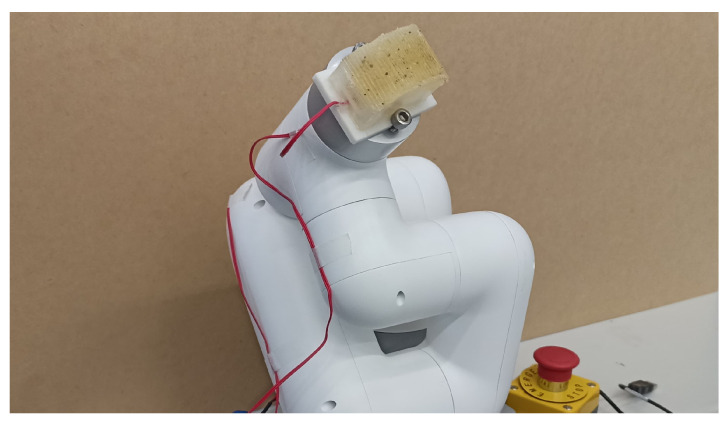
Sensor placement in the robot arm.

The sensor was affixed to a six-degree-of-freedom articulated robot arm (MyCobot, Elephant Robotics Co., Ltd., Shenzhen, China) as shown in Figure 6. The sensor slid over a 13 cm specimen at a constant speed of 30 mm/s, with the normal contact force maintained at 1 N. The sensor continually scanned the two specimens alternately, completing five iterations for each. The sensor then scanned a 13 cm long piece of sandpaper only once. During this durability test, the sensor exhibited slow abrasion damage. To accelerate the abrasion process, we introduced sandpaper (Sankyo Rikagaku Co., Ltd., Okegawa, Japan) with a 400-grit level. Once the scanning process started, the experimenter did not interfere with the measurement conditions.

The abrasion damage affected the output signals from the strain gauge, resulting in a decline in the signal-to-noise ratio (SNR). This SNR was calculated as described in Section 3.3. In this experimental setup, the scanning procedure was halted when the SNR level dropped below 15 dB for both types of specimens, although specific signal level requirements were not defined in this study. The abraded surface ridges were then reshaped through the method in Section 2.5. Subsequently, the specimens were slid with the sensor.

It is important to note that the surface of the sensor was greasy immediately after the manufacturing and healing processes. The surface was then cleaned and conditioned with talcum powder, and the sensor was slid over the specimen fifty times before the data collection process began. During this pre-conditioning process, the sensor and specimens were wiped several times with an industrial cloth.

### 3.3. Data Analysis

The epidermal ridges of the sensor exhibited dimensions larger than those of human epidermal ridges. Nevertheless, the sensor demonstrated the capacity to capture features on the specimen surface, as affirmed by the fundamental relationship that describes the spatio-temporal conversion of waves [36,37]:

(1) v=fλ,

where *v* is the sliding velocity (30 mm/s), *f* is the vibratory frequency (Hz), and λ is the spatial wavelength of the specimen (mm).

Given the signal amplification role inherent to the epidermal ridges [6], the abrasion of the ridges can be inferred from the signal levels. To assess the signal deterioration caused by abrasion, we utilized the SNR of the voltage signals. The SNR was calculated as follows:

(2) $$\mathrm{SNR}=20\;\log_{10}\left(\frac{V_{\mathrm{signal}}}{V_{\mathrm{noise}}}\right),$$

where $V_{\mathrm{signal}}$ and $V_{\mathrm{noise}}$ represent the amplitudes of the signal and noise, respectively. These amplitudes correspond to frequency components of 30 Hz and 15 Hz for specimens with spatial wavelengths λ = 1 mm and 2 mm, respectively. The noise level was determined from the sensor outputs when the robot moved in the air without contact with the specimens.

## 4. Results

**Figure 7:**
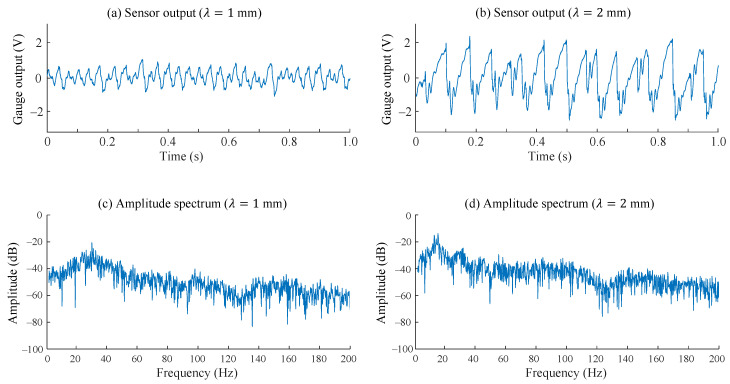
Examples of sensor outputs. Data from the 50th scan. Reference (0 dB) is 1.0 V.

The sensor tested specimens with spatial wavelengths (λ) of 1 mm and 2 mm at a velocity of 30 mm/s. Figure 7 shows examples of raw signals from the strain amplifier in volts and their corresponding amplitude spectra after removing the DC components. The amplitude spectra revealed peak frequencies of 30 Hz and 15 Hz, respectively, accompanied by harmonic peaks at multiples of these frequencies, namely 60 Hz for λ = 1 mm and 30 Hz for λ = 2 mm.

**Figure 8:**
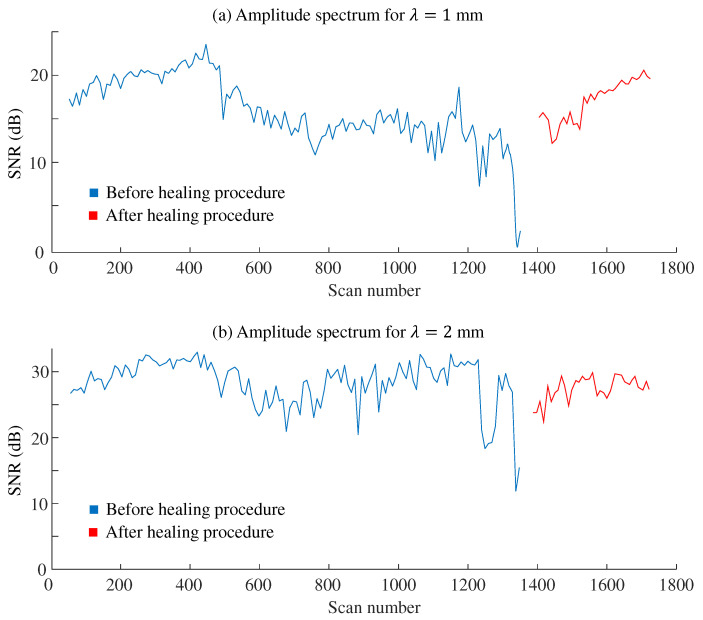
SNR for each specimen. Each point represents the mean acquired from five successive scans. The ridges were healed after the iteration of 1350 times. (**a**) SNR at 30 Hz signal components for the specimen of λ = 1 mm before and after healing. (**b**) SNR at 15 Hz signal components for the specimen of λ = 2 mm before and after healing. After the 1350 scans, the sensor’s ridges were reshaped. Fifty scans were performed to pre-condition the tactile sensor immediately after removal from the mold. The data during these processes were not recorded.

Figure 8 displays the SNR values as the scanning procedures were repeated. For the specimen with λ = 1 mm, the SNR values were nearly 20 dB before the 500th scan. The values then gradually, but not monotonically, decreased, reaching below 10 dB after the 1300th scan. For the specimen with λ = 2 mm, the SNR values were mostly above 25 dB before the 400th scan. With some fluctuations, the values then dropped below 15 dB after the 1300th scan. After the healing process, the SNR levels returned to those observed before the sensor experienced significant abrasion.

**Figure 9:**
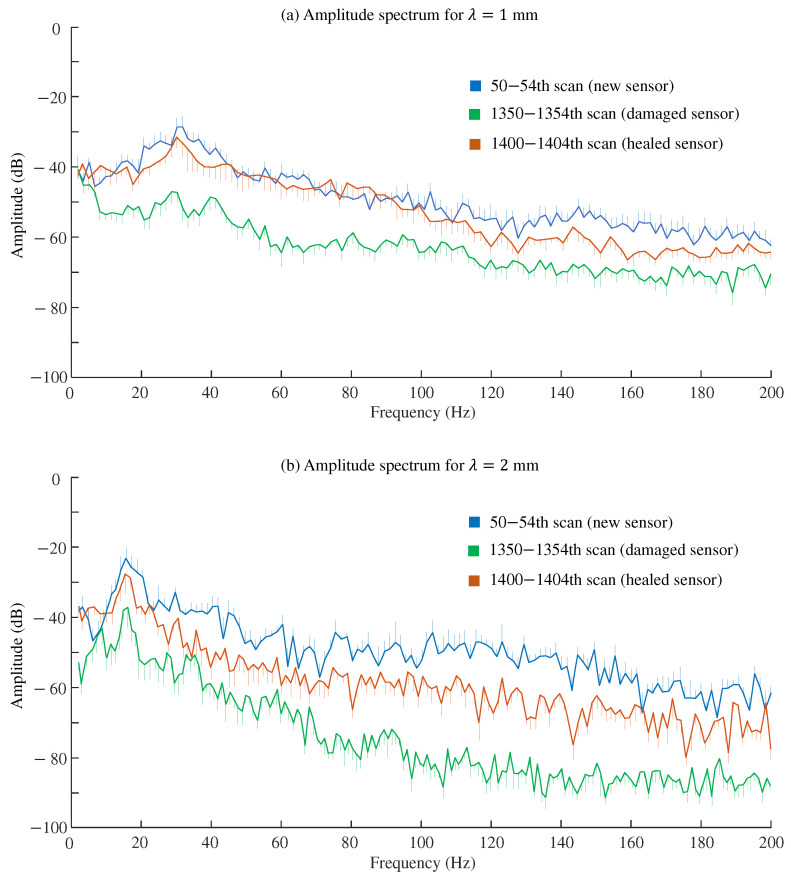
Comparison of amplitude spectra between new, damaged, and healed sensors. Means and standard errors across five successive scans. (**a**) λ = 1 mm. (**b**) λ = 2 mm. Reference (0 dB) is 1.0 V.

Figure 9 shows the amplitude spectra after the 50th scan (new sensor), 1350th scan (abraded sensor), and 1400th scan (healed sensor), with each representing the mean of five scans. Comparing the new (blue lines) and damaged (green lines) sensors, abrasion reduced the signal intensities across a wide frequency range of up to 200 Hz. As shown in Figure 9a for λ = 1 mm, the signal reduction of approximately 20 dB was observed across the range. In contrast, for λ = 2 mm shown in Figure 9b, the signal attenuation was nearly 30 dB in the range of 100–150 Hz. The decrease in amplitude after 1350 scans depended on the frequency and specimen’s λ value. Following the healing procedure (brown lines), the signals exhibited patterns similar to those of a newly manufactured sensor, although the amplitude levels of the healed and new sensors did not completely match. The amplitude levels of the healed sensor lagged behind those of the new sensor by approximately 20 dB at maximum, particularly in the 115–145 Hz range for the specimen with λ = 2 mm. Nonetheless, this frequency range was far from the main frequency, that is, 15 Hz, calculated by Equation (1).

**Figure 10:**
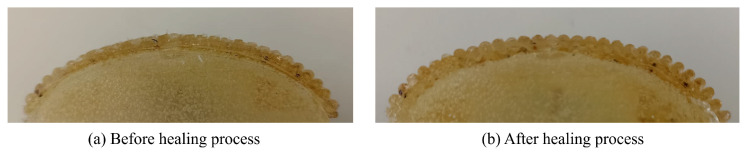
Sensor’s ridges. (**a**) Abraded sensor before the healing process. (**b**) Sensor after the healing process.

The damage of the epidermal ridges was visually observed as shown in Figure 10a. In contrast to an undamaged sensor, the affected sensor exhibited a flattening of the ridges. Figure 10b shows that the impaired ridges had been restored after the healing process.

## 5. Discussion

This study focused on restoring tactile texture sensors, particularly the segment mimicking epidermal ridges. These sensors suffer from abrasion damage after repeated use, and prior studies have not explored approaches similar to those examined in this study. Through experimentation, we observed how the SNR of the sensor deteriorated due to abrasion damage. Furthermore, these SNR values were partly restored after the healing process of the ridges.

The SNR is an assessment index based on a single frequency component and does not account for other components. As shown in Figure 9, abrasion may affect a wide frequency range. For evaluating such wide ranges, the evaluation indices used in previous studies [38,39,40] may be utilized. These studies assessed the quality of vibrotactile signals including multiple frequency components, taking into account human sensitivities to different frequency bands. Such indices might help in discussing how the tactile sensor’s performance is impacted by damage from iterative use and how it might be mitigated by the healing process. In the future, discussions should consider a wide frequency range, up to 1000 Hz [41], in relation to human sensitivity.

In the field of tactile texture sensors, there is no established standard for sensitivity levels. However, a threshold of 20 dB is typically considered indicative of a quality sensor in some fields, such as wireless communication [42]. Referring to this standard, as shown in Figure 8, the SNR dropped below 20 dB after 500 scans for λ = 1 mm, whereas the SNR remained above 20 dB even after 1000 scans for λ = 2 mm. One potential application of the tactile texture sensor is the autonomous classification of textures [43,44,45]. Therefore, the necessary sensitivity levels should be discussed in the context of textural classification in future studies. Furthermore, in actual industrial setups, the use of calibration specimens is recommended for monitoring the signal intensity levels. During the long-term and continuous use of the sensor, the calibration specimens should be periodically scanned by the sensor, and the amplitude spectrum of the signal outputs should be analyzed. If a problematic decrease in the response levels is detected, the user should be prompted to heal the sensor’s surface structure.

Some experimental results did not align with our expectations. We had predicted that, as the sensor underwent repeated use, abrasion would progress and the SNR would decrease monotonically. However, as shown in Figure 8, the SNR did not necessarily decrease monotonically with abrasion. In some parts, the SNR increased with repeated scanning. In other parts, the SNR fluctuated. One possible cause is the wear powder from the sensor material. Since the robot continued to scan the specimen automatically, the friction conditions during the experiment might not have been maintained due to the wear powder on the specimens. Furthermore, the sensor’s ridges were not only abraded but also flaked off during iterative use. Such sudden changes in the surface conditions might have led to abrupt changes in the sensory signals. Nevertheless, we do not have a convincing explanation for the irregular changes in the SNR with the number of scans.

The most significant remaining problem is that the frequency response of the sensor did not fully recover even after the ridges were apparently reshaped after the heating process. A plausible reason is that the condition of the material in the deeper region near the strain gauge was not fully restored. A potentially effective solution to this problem is to increase the temperature of the mold used to heal the sensor’s surface ridges. In this study, the temperature was set at 70 °C, which was determined to be sufficient for reshaping the ridged surface by visual inspection. Future work will focus on optimizing this process.

In the pursuit of our objective to restore sensor epidermal ridges, abrasive action was simulated using sandpaper. However, the real-world use scenarios may involve a variety of types of materials, necessitating further experimentation to assess the sensor’s durability under diverse operational conditions. Polyvinyl chloride plastisol was chosen as the primary material for the sensor due to its accessibility and capacity to reshape after heating. Despite its effectiveness, alternative healable materials such as microencapsulated isocyanates [21], poly(disulfides) [22], and natural rubber [23] offer the potential to enhance the capabilities beyond those of polyvinyl chloride plastisol. Therefore, continued experimentation with these healable materials is essential to optimize sensor performance.

## 6. Conclusions

This study explored the concept of a healable tactile texture sensor to address the problem of ridges’ abrasion. Earlier studies of healable materials developed soft robotic fingers or skins with sensing capabilities [17,18,19,20,25,26]. However, these studies have not addressed the abrasion of ridges unique to tactile texture sensors. Following the synthesis and molding of a prototype sensor, distinct capabilities were observed between a newly manufactured sensor and one with damaged ridges. The restoration of the damaged ridges demonstrated the ability to recover the sensor’s performance to nearly peak levels. As a future extension, it would be worthwhile to explore alternative healable materials and test the designed sensor across a wider range of textures.

## Data Availability

Dataset available on request from the authors.
